# Simulation-Based Rapid Development and Implementation of a Novel Barrier Enclosure for Use in COVID-19 Patients: The SplashGuard CG

**DOI:** 10.1155/2020/3842506

**Published:** 2020-12-17

**Authors:** Tine François, Laurence Tabone, Arielle Levy, Laurence Alix Seguin, Taher Touré, Carl Eric Aubin, Philippe Jouvet

**Affiliations:** ^1^Department of Pediatrics, Pediatric Intensive Care Unit, Centre Hospitalier Universitaire Sainte-Justine, Université de Montréal, Montreal, Canada; ^2^Department of Emergency Medicine, Centre Hospitalier Universitaire Sainte-Justine, Université de Montréal, Montreal, Canada; ^3^Department of Anesthesia, Centre Hospitalier Universitaire Sainte-Justine, Université de Montréal, Montreal, Canada; ^4^Department of Mechanical Engineering, Polytechnique Montréal, Montréal, Canada; ^5^Research Center, Centre Hospitalier Universitaire Sainte-Justine, Montréal, Canada

## Abstract

**Background:**

The current COVID-19 pandemic has resulted in over 54,800,000 SARS-CoV-2 infections worldwide with a mortality rate of around 2.5%. As observed in other airborne viral infections such as influenza and SARS-CoV-1, healthcare workers are at high risk for infection when performing aerosol-generating medical procedures (AGMP). Additionally, the threats of a global shortage of standard personal protective equipment (PPE) prompted many healthcare workers to explore alternative protective enclosures, such as the “aerosol box” invented by a Taiwanese anesthetist. Our study includes the design process of a protective barrier enclosure and its subsequent clinical implementation in the management of critically ill adults and children infected with SARS-CoV-2.

**Methods and Results:**

The barrier enclosure was designed for use in our tertiary care facility and named “SplashGuard CG” (CG for Care Givers). The device has been adapted using a multi- and interdisciplinary approach, with collaboration between physicians, respiratory therapists, nurses, and biomechanical engineers. Computer-aided design and simulation sessions throughout the entire process facilitated the rapid and safe implementation of the SplashGuard CG in different settings (intensive care unit, emergency department, and the operating room) during AGMPs such as bag-valve-mask ventilation, nasopharyngeal suctioning, intubation and extubation, and noninvasive ventilation. Indications for use and anticipatory precautions were communicated to all healthcare workers using the SplashGuard CG. The entire process was completed within one month.

**Conclusion:**

The rapid design, development, and clinical implementation of a new barrier enclosure, the “SplashGuard CG,” was feasible in this time of crisis thanks to close collaboration between medical and engineering teams and the use of recurring simulation sessions to test and improve the initial prototypes. Following this accelerated process, it is necessary to maintain team skills, monitor any undesirable effects, and evaluate and continuously improve this new device.

## 1. Introduction

The current COVID-19 pandemic caused by the severe acute respiratory syndrome coronavirus 2 (SARS-CoV-2) has resulted in over 54,800,000 cases of infection since its first description in December 2019 in Wuhan, China, and has already led to over 1,300,000 deaths worldwide [1]. It is estimated that approximately 6% of the infected cases have been healthcare workers [2]. Data from previous airborne viral infections, such as SARS-CoV-1 and influenza virus infections, suggest an elevated risk of aerosolization [3]. In addition, certain clinical situations and procedures may generate even more airborne aerosols [4, 5]. Invasive procedures such as endotracheal intubation or the use of noninvasive ventilation in COVID + patients represent particular risks while air is insufflated into the oropharynx under positive pressure with the possibility of air leaks. These aerosol-generating medical procedures (AGMP) put healthcare workers at even greater risk for infection, especially in the critical care setting. Risks related to aerosolization are so important that initial recommendations for adult patients were to avoid noninvasive ventilation and proceed directly to intubation in COVID-positive patients with emerging respiratory distress [6]. However, there is a higher mortality rate in immunocompromised pediatric patients when invasively ventilated. Furthermore, the use of noninvasive ventilation in the adult population with COVID has now been proven to reduce the need for intubation [7]. Children are at higher risk of complications related to intubation, such as subglottic stenosis [8]. Thus, early intubation of COVID + pediatric patients without a prior trial of noninvasive support is not acceptable for pediatric critical care physicians.

For hospitalization of a COVID-infected patient, it is recommended to admit them to a negative-pressure room and for healthcare providers to wear standard personal protective equipment (PPE), namely, gown, gloves, and an N95 mask. Access to the standard PPE could become limited because of the high global demand, interrupted transportation lines, and altered deliveries in many countries including Canada [9]. This concern motivated clinicians to develop other protective devices to minimize aerosol exposure for healthcare providers during high-risk AGMPs. In March 2020, a Taiwanese anesthetist released the first design for a device that aimed to protect physicians during the endotracheal intubation of a COVID-positive patient [10]. Several weeks later, Canelli et al. published a video in which the use of the “aerosol box” as a protective barrier enclosure during endotracheal intubation showed no macroscopic contamination outside the box [11]. Protection against aerosolization is less clear, however, and there is probably an increased risk for infection by removing the box directly following an intubation [12].

We devised a similar protective barrier enclosure at our tertiary care facility and named it “SplashGuard CG,” CG for Care Givers [13]. The SplashGuard CG has been adapted in such a way that it can easily be applied and used in children or adults and in multiple settings and situations as will be detailed further in the article.

In this report, we aim to describe in detail the accelerated process of design and implementation of this new open innovation technology, with particular emphasis on the role of simulation in its development. We will also share the challenges encountered throughout this process, which took place at the very beginning of the COVID pandemic.

## 2. Methods

**Figure 1:**
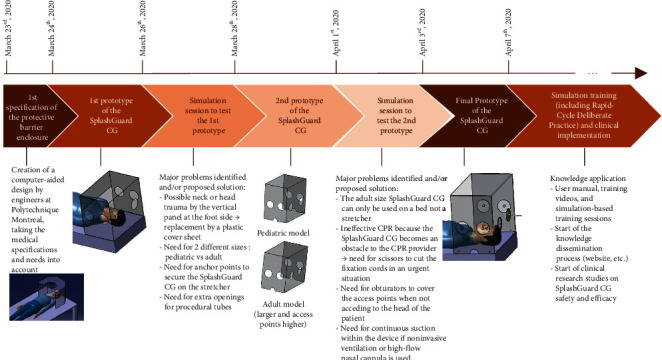
Main steps in the design and implementation of the SplashGuard CG.

The aim of the device development was to create a protective barrier enclosure for care givers, to be used in combination with standard PPE, for protection against contact and airborne transmission from pediatric and adult proven or suspected COVID patients. Its development and implementation used a living lab methodology including prototyping by engineers and testing in simulated conditions by end users (see Figure 1):

1. The prototype design took place in the technical laboratory using computer aided design (CAD), with the collaboration of a pediatric intensivist and a biomechanical engineer.
2. The simulation testing was performed in a simulated clinical environment. The prototype was tested using simulations of various clinical situations including an intrahospital patient transport, installation of noninvasive ventilation, and endotracheal intubation using pediatric and adult manikins. A select interdisciplinary team (see Section 2.1 research team) discussed and reached consensus on the practical use of the SplashGuard CG, its technical characteristics and dimensions, and the various clinical situations where the use of the SplashGuard CG could be applicable. These interactions were video recorded for subsequent analysis of the feasibility of the device and its practical use.
3. After finalizing the prototype, the research team also identified the various risks and the safety measures that needed to be addressed when using the device and developed training sessions using Rapid Cycle Deliberate Practice (RCDP) to rapidly and efficiently train healthcare providers from the pediatric emergency, anesthesia, and pediatric critical care sectors. Rapid Cycle Deliberate Practice is a simulation-based instructional strategy that focuses on rapid acquisition of necessary skills when training for emergency clinical situations [14].
4. During the final phase, we established cleaning and disinfection procedures [15] and obtained legal advice according to Health Canada regulations for technology dissemination [16].

### 2.1. Research Team

The development team included biomechanical engineers from Polytechnique Montreal, simulation specialists from the Mother-Child Simulation Center at the Centre Hospitalier Universitaire Sainte-Justine, physicians from the pediatric intensive care unit and the emergency and anesthesiology departments, as well as respiratory therapists and nurses. In addition, refining of this new technology required further collaboration with biomedical engineering, the infection prevention unit, and the ergonomic units at our institution (see SplashGuard CG Study Group).

## 3. Results

### 3.1. Design and Main Objective of the SplashGuard CG Prototype

Starting on March 23, 2020, the SplashGuard CG was designed and developed at the Centre Hospitalier Universitaire Sainte-Justine in Montreal, Canada. The main objective during the development of the SplashGuard CG was to ensure safety and protection of healthcare workers managing a critically ill COVID patient, suspected or confirmed, who is in respiratory distress and requires aerosol-generating medical procedures, while equally respecting patient safety. Indications for use of the SplashGuard CG were established for hospitalized and bedridden patients with a highly contagious airborne infection, e.g., SARS-CoV-2. The use of the SplashGuard CG is suggested for (1) procedures at the head of the patient: intubation or extubation procedures, the placement of a oro- or nasogastric tube, oro- or nasopharyngeal aspiration, and installing the patient on high-flow nasal cannula or noninvasive ventilation and (2) patients admitted and supported by high-flow nasal cannula, noninvasive ventilation, or invasive ventilation (following the intubation procedure or tracheostomy), during transport or during their stay in one the various departments (ED, OR, and ICU).

### 3.2. Final Prototype Description

**Figure 2:**
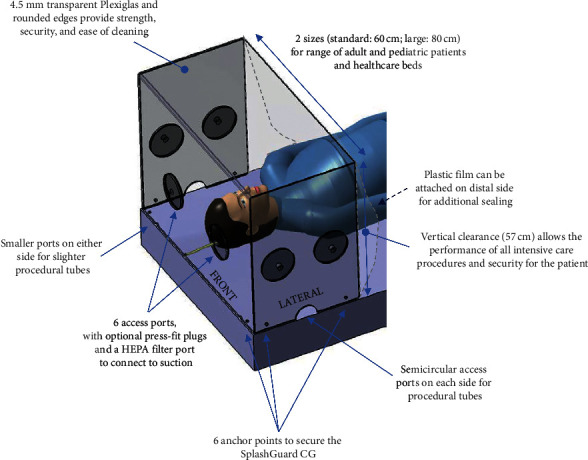
Features of the final prototype of the SplashGuard CG.

Following simulation sessions, the SplashGuard CG was developed and manufactured in 2 sizes: a larger and a standard model (Figures 1 and 2). The larger model is for use in an adolescent or adult admitted to the intensive care unit, the standard model for use in a pediatric patient admitted to the intensive care unit or for use in a pediatric or adult patient on a stretcher or an operating table. The SplashGuard CG contains an upper protection panel, a front-end panel with direct access to the patient's head through two large circular openings, and two large circular openings on each side panel. Because of the presence of six different access ports, several caregivers can gain access to the head of the patient simultaneously, one person on each of the three sides of the SplashGuard CG placed on the bed (head, right, and left sides). There is one semicircular opening at the bottom of each side as well as two smaller semicircular openings on the front panel to be able to introduce the respirator circuit and the suction equipment into the device, as well as the oxygen tubing from the Ayres bag-valve device or other self-inflating ventilation bag. These semicircular openings allow the SplashGuard CG to be removed in case of emergency without disturbing the position of all the tubes and equipment. There are six small holes (2 at the front panel and 2 on either side) that serve as anchor points: ties can be woven through each hole to attach and secure the SplashGuard CG to the hospital bed, operating table, or stretcher.

Following a first simulation session with the initial prototype, the size and height of the box were adjusted to the space needed for providers to perform different aerosol-generating medical procedures in a patient with a suspected or confirmed SARS-CoV-2 infection. We also adjusted the position of the access ports to ensure successful and safe assistance and completion of AGMPs. The vertical panel at the distal end of the SplashGuard CG, present in the first prototype, was changed for a plastic cover sheet attached to the SplashGuard CG to avoid patient neck or head injury when the patient wants to sit up.

Anticipatory precautions when using the SplashGuard CG were also identified and further detailed during the whole process. There are theoretical risks of asphyxia, hypercarbia, impaired care during emergency situations, injury, and pressure wounds, which we will describe in detail in the following section.

(1) Risk of asphyxia: the SplashGuard CG cannot be used in a unit where the patient is not under continuous surveillance because of the risk of acute asphyxia associated with the use of soft plastics close to the face. Thus, it is mandatory that the SplashGuard CG be used in a setting offering continuous patient monitoring, such as the intensive care unit, the emergency department, or the operating room. (2) Risk of hypercarbia: if the SplashGuard CG is used in a patient who is breathing spontaneously, a theoretical risk of hypercarbia exists if the gas flow entering the SplashGuard CG is insufficient. (3) Other risks attributable to emergency situations: there are conditions that require the immediate removal of the SplashGuard CG such as cardiac arrest, but access to the patient could be delayed by its presence. For that reason, a pair of scissors is attached to the top of the box at all time to cut the fixation ties if necessary. (4) Risk of injury: it is necessary to secure the SplashGuard CG to the hospital bed or stretcher, especially in cases of prolonged use, transport of a COVID-patient between units or when caring for agitated pediatric patients, by using the anchoring points designed for that purpose. (5) Risks related to pressure wounds: the possible risk of developing a pressure wound due to immobilization was also addressed in the instruction manual, with areas at risk identified as the scalp, shoulders, and arms of the patient.

### 3.3. Refinement of SplashGuard CG Use for Different Procedures

After elaborating the final prototype, the device was once again tested for use during a second simulation session (Figure 1). Following this session, the different clinical situations and various procedures that were identified for SplashGuard CG use, in COVID-suspected or COVID-confirmed patients, were confirmed to be transport of a patient with respiratory symptoms through hospital “cold” zones, endotracheal intubation or extubation of a patient, installation and maintenance of noninvasive ventilation, nasopharyngeal aspiration, bag-valve-mask ventilation, tracheostomy care, and the installation of a laryngeal mask or a nasogastric tube.

The use of the SplashGuard CG is also recommended when invasive positive-pressure ventilation is maintained, at the discretion of the healthcare team, especially when considering the use of prone positioning. When use of an enclosed system for endotracheal suctioning is not possible, risks of aerosolization are increased because it can trigger cough, so the SplashGuard CG can also be used in this context.

To ensure protection against the aerosolization of viral particles when no AGMPs are performed, six dispensable plastic obturators for the large circular access ports were created, in addition to the use of a plastic transparent shield on the open side of the device which allows easy access to the patient at all times. When using the SplashGuard CG in the intensive care unit, one or two of the obturators at the front panel contain an opening to connect to the wall suction to create a negative-pressure environment within the box and limit aerosolization of viral particles.

The use of the SplashGuard CG for caregiver protection, when supporting patients with noninvasive positive-pressure ventilation or high-flow nasal cannula, requires the availability of continuous aspiration in the device, by using the obturators as described before. For short procedures (intubation, extubation, etc.), continuous aspiration is not recommended in order to allow the provider and those assisting him full access to the patient's head.

Performance of a simulated cardiac arrest requiring cardiopulmonary resuscitation (CPR) demonstrated the limitations imposed by the SplashGuard CG as to proper hand positioning and posture during CPR and delays in defibrillator pad placement. A recommendation was added in the instruction manual to promptly remove the SplashGuard CG, by cutting the ties with the scissors, in case of an acute emergency or need for CPR.

After validation by the infection prevention unit, an arbitrary recommendation was made to change the SplashGuard CG every 24 hours in cases requiring high-flow nasal cannula or noninvasive ventilation and every 48 hours in other cases to avoid the adherence of viral particles to the inner surface of the device. This recommendation provides an appropriate time window for disinfection of the device with 0.5% hydrogen peroxide [15].

### 3.4. Training of the Healthcare Teams

An instruction manual, including a full description of the various predefined clinical scenarios, was edited for education and training of all members of the emergency, critical care, and anesthesia healthcare teams.

**Figure 3:**
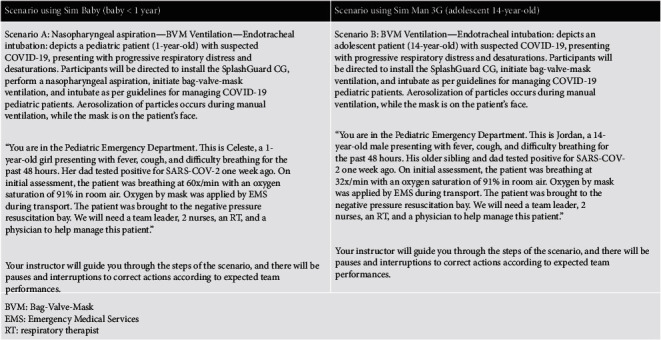
Brief description of the clinical scenarios used during simulation.

Prior to implementing the SplashGuard CG within the clinical environment, we organized several clinical-based simulation sessions and used Rapid Cycle Deliberate Practice to train healthcare providers from the pediatric emergency, anesthesia, and pediatric critical care departments. This training program consisted of various simulation scenarios (see Figure 3) for an interprofessional group of healthcare workers, including a physician, a nurse, and a respiratory therapist at each session. Simulation sessions were first conducted at the simulation center followed by in situ training in the respective clinical environments, i.e., pediatric intensive care unit (PICU), emergency department (ED), and operating room (OR). A written user manual as well as training videos explaining how to use the SplashGuard CG during different procedures were distributed by e-mail to the participants and made accessible on a website [13]. During the first few weeks after the final prototype development, we were able to train 71 healthcare workers: 61 physicians, respiratory therapists, and nurses were trained in the simulation center, and 10 healthcare workers were trained during in situ simulation sessions in the ED.

### 3.5. Clinical Implementation of the SplashGuard CG and Further Evaluation

The SplashGuard CG was first authorized for clinical use in the PICU, the OR, and the ED, on April 7^th^, 2020, three weeks after the start of the innovation process. The PICU staff agreed to provide noninvasive ventilatory support and high-flow nasal cannula for COVID-suspected and COVID-positive patients, if the SplashGuard CG was available. This eliminated the need for systematic intubation in several patients. The SplashGuard CG has also been used routinely in the ED for COVID-suspected or COVID-confirmed patients with respiratory symptoms to transport patients from the ED to the PICU and in the operating room for multiple intubation and extubation procedures.

Several clinical research studies, evaluating the safety and efficacy of the SplashGuard CG, were developed in April 2020 and are still ongoing, in addition to the continuous evaluation by the different medical teams.

## 4. Discussion

The rapid development and implementation of a secure protective barrier enclosure, including clinically relevant features, were accomplished by a diverse research team including engineers, caregivers, and several other experts, in the context of the COVID-19 pandemic. The well-elaborated plan, ensuring close collaboration between interprofessional teams from different departments, and using Rapid Cycle Deliberate Practice for intensive simulation training, contributed to the success of this process. A user manual, instructional videos, and both laboratory and in situ simulations made the rapid development and implementation of our SplashGuard CG successful in less than a month.

We acknowledge that there were several challenges encountered during the process. Multidisciplinary and interdisciplinary discussions on a regular basis were a key component to making this accelerated process succeed. However, finding time to meet in times of crisis with the necessary social distancing despite every team member having a full schedule was a major challenge to the process. There definitely was a need for a team leader to facilitate the planning and coordination of this process and to encourage every team member to participate. In addition, the rapidly evolving knowledge on the characteristics of SARS-CoV-2, as well as the experience of other centers implementing similar barrier enclosures, had to be taken in account during the development process.

In a recent commentary in the New England Journal of Medicine, Rosenblatt et al. criticized the implementation of the “aerosol box” barrier enclosure described by Canelli [11, 17]. Equally, several limitations of the Taiwanese “aerosol box” have been highlighted. Addressing these limitations, as described below, has been a priority. (i) Freedom of hand and arm movement: The box was adapted and enlarged to ensure that hand and arm movements would not be restricted when performing intubation or other procedures demanding placement and access to the head of the patient. We can attest to the need for simulation training to adapt the hand and arm movements inside the SplashGuard CG. However, the providers reported a quick and easy learning curve when performing various procedures with the SplashGuard CG in place. They also reported that there is enough space within the SplashGuard CG to place the equipment needed for the different procedures as well as enough access to the head of the patient. Respiratory therapists in our pediatric hospital became quickly at ease with the use of the SplashGuard CG, as many of them are used to providing care to neonates in incubators in which the access to the patient is similarly restricted. (ii) Access for multiple caregivers: By adding 2 extra access ports to each side of the device, for a total of six access ports, we created the opportunity for multiple caregivers to gain access to the patient at the same time while remaining protected from aerosolization. (iii) Ease of handling: While the SplashGuard CG is big, it is not heavy and can easily be carried by a single individual. It is, however, preferable that at least two people handle the device for its installation on a stretcher or hospital bed to be able to attach it securely to minimalize the risk of injury to the patient and the healthcare workers. (iv) Malposition due to patient agitation: Placing a half-open box over the head of a dyspneic patient may cause agitation, especially in patients with delirium, which could complicate the installation of the SplashGuard CG and interfere with patient care. We created fixation features to secure the SplashGuard CG to the bed to avoid accidents due to patient agitation. However, anticipating the need for urgent removal of the SplashGuard CG in critical situations, a pair of scissors to cut the ties remains attached to one of the sides. (v) Limitation of protection to healthcare providers: It cannot be overstated that while the SplashGuard CG may offer additional protection to healthcare providers, especially during AGMPs, it does not replace the standard PPE recommended during aerosolizing procedures. (vi) Prone positioning: We agree that for patients with acute severe respiratory distress syndrome in which the prone position becomes preferable, appropriate care for the patient could be delayed or hindered by the installation or removal of the SplashGuard CG. Healthcare workers should reflect carefully on the suitability of retaining the SplashGuard CG in this situation.

The question remains as to what degree the SplashGuard CG can protect against viral aerosols in addition to droplet protection. By attaching the plastic shield at the open side of the device, we avoid aerosols and droplets being redirected to the distal end of the bed, but as the plastic shield must be moved partially at some point in time to allow adequate patient care, there may be some exposure to the provider manipulating the plastic shield. For this reason, we made it possible to aspirate air within the SplashGuard CG to reduce contamination risks, as recommended by the FDA since August 2020 [18]. If the SplashGuard CG is used in a patient supported with high-flow nasal cannula, continuous aspiration can also resolve the issue of the plastic surface of the device becoming too fogged because of the warmed air. In doing so, adequate continuous visual surveillance of the patient is made possible.

The need for cleansing and disinfection of the device after use is mandatory, as all material and equipment in a patient's room is considered contaminated. We do not see this as a limitation, but precautions must be taken during disinfection as disinfection can create aerosolization.

## 5. Conclusions

This study demonstrates that the accelerated design, development, and rapid implementation of a new device is feasible in times of crisis through close collaboration between medical and engineering teams and using repeated simulation sessions to test and improve the initial prototypes. Once the final prototype of our barrier enclosure “SplashGuard CG” was developed, the rapid dissemination of knowledge to all healthcare workers involved simulation-based training, including Rapid Cycle Deliberate Practice, and the use of supplementary means such as written user manuals, a website, and training videos. This simulation-based development and implementation of a new device seems particularly crucial during a pandemic situation.

It should, however, be highlighted that following this rapid process, particular attention must be paid to maintaining team skills, monitoring the global evaluations of users, and any undesirable effects, all of which will contribute to continuous improvement of the device.

## Data Availability

No data were used to support the findings of this study.
